# Adapted Helping Babies Breathe approach to neonatal resuscitation in Haiti: a retrospective cohort study

**DOI:** 10.1186/s12887-021-02987-4

**Published:** 2022-01-03

**Authors:** Shannon Findlay, Morgan Swanson, Christian Junker, Mitchell Kinkor, Karisa K. Harland, Christopher Buresh

**Affiliations:** 1 Department of Emergency Medicine, University of Iowa, Roy J. and Lucille A. Carver College of Medicine, 200 Hawkins Drive, IA 52242 Iowa City, USA; 2 Department of Emergency Medicine, University of Washington, Harborview Medical Center, Box 359702, 325 Ninth Avenue, WA 98104 Seattle, USA

**Keywords:** Helping babies breathe, Training, Community health workers, Neonatal, Birth asphyxia

## Abstract

**Background:**

Helping Babies Breathe (HBB) is an American Academy of Pediatrics neonatal resuscitation program designed to reduce neonatal mortality in low resource settings. The 2017 neonatal mortality rate in Haiti was 28 per 1000 live births and an estimated 85 % of Haitian women deliver at home. Given this, the Community Health Initiative implemented an adapted HBB (aHBB) in Haiti to evaluate neonatal mortality.

**Methods:**

Community Health Workers taught an aHBB program to laypeople, which didn’t include bag-valve-mask ventilation. Follow-up after delivery assessed for maternal and neonatal mortality and health.

**Results:**

Analysis included 536 births of which 84.3 % (n=452) were attended by someone trained in aHBB. The odds of neonatal mortality was not significantly different among the two groups (aOR=0.48 [0.16-1.44]). Composite outcome of neonatal health as reported by the mother (subjective morbidity and mortality) was significantly lower in aHBB attended births (aOR=0.31 [0.14-0.70]).

**Conclusion:**

This analysis of the aHBB program indicates that community training to laypersons in low resource settings may reduce neonatal ill-health but not neonatal mortality. This study is likely underpowered to find a difference in neonatal mortality. Further work is needed to evaluate which components of the aHBB program are instrumental in improving neonatal health.

**Supplementary Information:**

The online version contains supplementary material available at 10.1186/s12887-021-02987-4.

## Background

Neonatal mortality is a significant global health concern [1]. Despite great strides, there are still wide disparities around the globe. In 2017, the neonatal mortality rate in Haiti was 28.3 per 1000 live births [2]. 25 % of these were thought to be due to asphyxia and birth trauma [3]. Helping Babies Breathe (HBB) is a program that has shown significant success in lowering rates of still births by 24 % and early neonatal deaths by 47 % in low resource settings when healthcare workers are trained [4].

The key concept in HBB is *The Golden Minute* which identifies the steps a birth attendant must take immediately after birth to evaluate the baby and stimulate breathing [5]. An estimated 3-6 % of neonates will require bag-valve-mask (BVM) ventilation and less than 1 % will require more advanced methods of resuscitation [5].

Based on the widespread success of HBB, we taught a modified HBB program in coordination with a not-for-profit organization, Haiti Community Health Initiative (CHI). CHI runs regular “pop up” clinics in villages in the mountains east of the Arcahaie region. They host clinics in village common areas every 2-3 months throughout the year. As of 2017, only 42 % of deliveries in Haiti had a skilled birth attendant present [6]. Within the rural population CHI serves, only 14 % of women deliver in a hospital. The majority who deliver at home don’t have a trained attendant present. Rather, these women were delivered at home by relatives or neighbors without any sort of training or equipment. The women choose to deliver at home because of concerns about the cost of care, the distance to a hospital, and the perception that the care received is poor [7]. While there is widespread agreement it is better for women to deliver in a hospital where medical expertise is available, for some populations there will need to be widespread reductions in systematic barriers before that becomes a reality.

Typically, HBB program is performed within a healthcare setting like a clinic or hospital, but it has also been delivered successfully by community health workers (CHWs) in populations that lack access to health care in low- and middle-income countries. This population in Haiti lives in a very remote area with no access to healthcare and limited access to CHWs, so we performed an intervention with laypeople. These laypeople are persons who often live in close proximity to the pregnant women. They are often relatives, but sometimes friends or domestic partners, and traditionally they are the ones who actually attend the deliveries. These participants had no formal health education, and in fact 47 % of them had self-identified as illiterate in a 2013 survey [7]. The deliveries in this study were all performed at home.

Since only a small percentage of neonates require BVM ventilation we omitted it from the training. BVM is a clinical skill that requires necessary training to master to avoid injury [8, 9], and we were training laypeople who will not have a chance to practice regularly. This pilot study is the first time this training has been conducted with a group of laypeople with varying degrees of education and literacy and has been implemented in a rural home-birth setting. Our aim was to implement aHBB among this population to reduce neonatal mortality and ill-health.

## Materials and methods

### Study design & setting

This is a retrospective cohort study of the implementation of a lay neonatal resuscitation program in the Arcahaie region of Haiti from July 2015 to May 2019. Data were collected by CHWs working for CHI as part of a quality improvement process.

### Description of intervention & study population

CHI has been holding pop up clinics in the area since 2011. CHWs are people selected by the villages they serve who have undergone 6 months of basic health and health education training. They visit each house in their village on a monthly basis to assess individual health, follow up on people with chronic diseases, and provide health education. All CHWs underwent HBB Training [10] at a train-the-trainer session. They were also provided with teaching materials for reference and mannequins at this training and again 2 years later. The program run by the not-for-profit organization was modified to omit BVM ventilation due to concerns about the difficulty of having a lay population learn a complex and potentially dangerous intervention. The CHWs then taught the aHBB programs to the lay population.

A convenience sample of participants were recruited among women coming through clinic in the second half of their pregnancy, after 20 weeks by last menstrual period. Additional participants were recruited as the CHWs checked on the population who they are charged for looking after. All women were given clean birth kits that included soap, gloves, clean blankets, a clean razor, string, and a bulb suction syringe, were given counseling on birth preparedness, cord care, and were given teaching on the importance of early and exclusive breast feeding, whether or not they chose to participate in the program. They were all shown how to suction the mouth and nose of a baby and how to use the string and razor to tie off and cut the umbilical cord. All of these women were offered enrollment into the aHBB program, and informed verbal consent was obtained from participants after explaining what participation would entail. Verbal consent was utilized due to the high rates of illiteracy in the population served. Women who consented to participate self-selected participation in the aHBB program. If they chose to participate in the aHBB program, they were asked to identify the person who would assist them at delivery. These assistants were offered three separate trainings of the aHBB curriculum. The curriculum’s focus is on basic neonatal resuscitation techniques within the first minute of life: stimulation, drying and warmth, and bulb suction. There are twelve components to the curriculum (Table 1). Each session was followed by a test resuscitation on a mannequin to ensure uptake of skills and knowledge. The goal was to have the assistant complete three separate trainings by the CHWs before delivery of the baby. CHWs underwent skills refresher training every other year.

**Table 1:** Description of the 12 aHBB Training Components

Step	*Performed*	*Definition*
Preparation	yes no	Get yourself, your supplies, and the environment ready for the delivery
Wash your hands and put on gloves	yes no	Wash your hands thoroughly with soap and clean water and put on gloves
Find clean flat place	yes no	Find a place to make supplies ready and place the baby after delivery
2 clean dry sheets	yes no	Get clean dry sheets for delivery
Sterile razor	yes no	Get a sterile razor to cut the umbilical cord
2 Pieces of string	yes no	Get two pieces of string to tie the cord
Suction	yes no	Get bulb suction to suction secretions
Dry baby and remove wet cloth	yes no	Dry baby and remove wet cloth
Suck liquid from the mouth then nose	yes no	Using the bulb suction, suck liquid from the baby’s mouth then nose if they are not crying
Stimulate baby if it doesn’t cry	yes no	If the baby is not crying stimulate the baby
Tie the cord at the mother and the baby	yes no	Tie the cord in two places near the baby’s umbilicus
After 3 min, cut the cord	yes no	Cut the cord between the ties after three minutes

### Follow-up

CHWs followed up with the mother after the birth to understand how the delivery went and ascertain the health status of the mother and baby. The birth attendants were not interviewed. The date of follow-up, feedback about events at delivery, self-reported training status of the attendant, and self-reported health status of the mother and baby were recorded in the database. The date of follow up was not standardized, as it sometimes required distant travel to reach participants, which was complicated by fluctuating fuel prices and occasional political instability. Feedback based on review of the records and participant comments were shared with the CHWs and de-identified data on neonatal and maternal outcomes was shared with the community at large.

### Data management

Attendance, dates of training, and performance on the post-training tests were recorded in a purpose made database created from Filemaker (version 14, Claris International Inc., Santa Clara, CA). Data were collected on individual electronic tablets and then were transferred via file export to a centralized spreadsheet that was de-identified and analyzed. Data were monitored periodically, and feedback was given to the CHWs and the community at large about outcomes.

### Ethical review & reporting

This study was approved by the University of Iowa Institutional Review Board. All of the experimental protocols involving human data were in accordance with the guidelines of University of Iowa Institutional Review Board. The study is reported according to the Strengthening the Reporting of Observational studies in Epidemiology guidelines [11].

### Key measures & covariates

The primary exposure was aHBB training status. aHBB training status was defined by self-report in response to the question “Was the birth attendant trained in HBB?” on the follow-up interview with the mother. Covariates ascertained from the follow-up interview included multiple gestations (continuous), birth attendant present at delivery (yes/no), and aHBB supplies ready on time (yes/no). Each element of the aHBB training at delivery was recorded as performed or not performed (yes/no) at the time of birth. Other variables considered were the number of births previously attended by attendants (continuous, collected from birth attendants upon enrollment). We were not able to collect data reliably about gestational age or weight at birth. Participants with missing primary outcome status or missing primary exposure status were excluded from the study.

### Outcomes

The primary outcome was neonatal mortality. Neonatal mortality was defined as any child who had died at the time of follow up by the CHW. Secondary outcomes included maternal mortality and self-reported morbidity, and the composite outcomes of morbidity or mortality reported for the neonate and the mother. These outcomes were defined *a priori*. Sample size calculations were not done, as this was a novel intervention and the pragmatic nature of the intervention, which allowed participants to select whether they wanted to be in the intervention group or the control group, made this unfeasible. All outcomes were ascertained by the CHWs interviewing the mother at the time of follow-up. Neonatal and maternal health were recorded as healthy, unhealthy, dead, or unknown. The terms ‘healthy’ and ‘unhealthy’ were not defined for the mother who was reporting on her condition and that of the neonate. This was a subjective report that was felt to be more patient-centered.

### Data analysis

Descriptive statistics and univariable tests of association, including chi-square and Wilcoxon-Mann-Whitney tests, were used to compare study subject demographics among the aHBB trained and untrained cohorts. The main exposure was self-reported aHBB training status recorded at the time of the follow-up survey. Unadjusted logistic regression initially estimated the association between aHBB training and neonatal and maternal mortality and ill health/mortality. Multivariable logistic regression models were constructed using a combination of purposeful selection, guided by previous literature and clinical knowledge, as well as backwards selection guided by Akaike Information Criterion (AIC). Additional uni- and multivariable logistic regression models estimated the odds of the four clinical outcomes by other measures of optimized care delivery, including aHBB supplies ready at birth, birth attendant at delivery, self-report of attending all aHBB trainings, and number of births attended (by birth attendant). Statistical analysis was conducted in SAS (version 9.4; SAS Institute, Cary, NC).

### Missing data

Demographics of the study cohort and the participants with missing data were compared to assess if there were observed differences in the study populations using descriptive statistics and univariable tests, chi-square and Wilcoxon-Mann-Whitney tests.

### Sensitivity analyses

Two sensitivity analyses were performed to test the robustness of study results to different definitions of aHBB training status. First, aHBB training status was redefined as recorded attendance at all aHBB training sessions with all other participants classified as not trained. Second, aHBB training status was defined as any training (self-report or recorded attendance for at least one aHBB training session) with only participants with no training (self-reported no training and no recorded attendance at training sessions) classified as not trained. Similar uni- and multivariable logistic regression techniques, as described above, were used to estimate odds of the four clinical outcomes.

## Results

### Description of study population

**Fig. 1:**
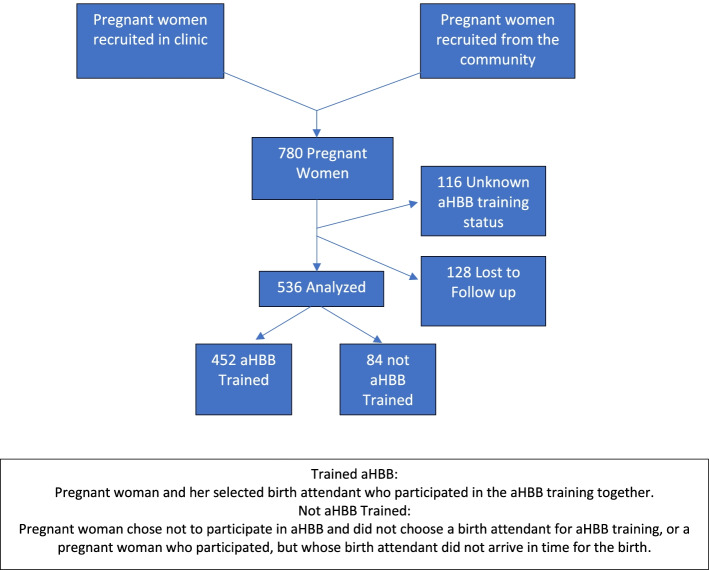
Flowchart of Study Participants

There were 780 mother-birth attendant pairs who consented for the study. Of the pairs, 116 had an unknown aHBB training status, and 128 participants (19.3 %) were lost-to-follow-up (i.e. had a missing outcome data) resulting in an analysis sample of 536 mother-layperson attendant pairs (Fig. 1). The majority (84.3 %) of the study population had aHBB training. Most had a singleton birth, and most attendants had attended no or few previous births (Table 2). The aHBB training group was more likely to have a birth attendant at delivery (93.1 % vs. 34.5 %, p<0.001), have aHBB supplies ready on time (92.3 % vs. 39.3 %, p<0.001), and more likely to have each of the 12 aHBB elements at delivery (p<0.001). There were no significant differences in reported demographics (number of births, births attended, birth attendant present, HBB supplies ready) or outcomes (neonatal or maternal) between the study cohort and the population excluded for missing exposure or outcome data (Table S1 ).

**Table 2:** Birth characteristics by training status

	Trained aHBB		Not Trained aHBB		
	**N= 452**		** N= 84**		
Variable	n	%	N	%	p-value
**Singleton vs. Multiple Gestation Birth**					0.94
1	419	92.7	77	91.7	
2	10	2.2	2	2.4	
Missing Data	23	5.1	5	6.0	
**Births Attended**, median (IQR)	0	(0-3)	0	(0-1)	0.20
**Birth Attendant Attending Delivery**					<0.001
Yes	421	93.1	29	34.5	
No	16	3.5	52	61.9	
Missing Data	15	3.3	3	3.6	
**aHBB Supplies ready on time**					<0.001
Yes	417	92.3	33	39.3	
No	11	2.4	32	38.1	
Missing	24	5.3	19	22.6	
**Neonatal Health**					0.05
Good Health	392	86.7	64	76.2	
Ill Health	29	6.4	11	13.1	
Dead	28	6.2	9	10.7	
Unknown	3	0.7	0	0.0	
**Neonatal Mortality**					0.14
Yes	28	6.2	9	10.7	
No	424	93.8	75	89.3	
**Maternal Health**					0.37
Good Health	398	88.1	71	84.5	
Ill Health	46	10.2	10	11.9	
Dead	2	0.4	0	0.0	
Unknown	6	1.3	3	3.6	
**aHBB at Delivery**					
Dry Baby and Remove Cloth	347	76.8	7	8.3	<0.001
Find Clean Flat Place	353	78.1	8	9.5	<0.001
After Three Minutes, Cut Cord	339	75.0	7	8.3	<0.001
Preparation	350	77.4	8	9.5	<0.001
Sterile Razor	350	77.4	7	8.3	<0.001
Stimulate Baby (if no crying)	346	76.6	7	8.3	<0.001
Suck Liquid from Mouth then Nose	345	76.3	7	8.3	<0.001
Suction	348	77.0	7	8.3	<0.001
Tie Cord	344	76.1	7	8.3	<0.001
Two Pieces of String	348	77.0	7	8.3	<0.001
Clean, Dry Sheets	352	77.9	7	8.3	<0.001
Wash your hands and put on gloves	353	78.1	8	9.5	<0.001

### Main results: HBB training and clinical outcomes

Neonatal mortality was similar in the aHBB trained and not trained groups (6.2 % vs. 10.7 %, p= 0.139), and there remained no difference in neonatal mortality by aHBB training status after adjustment (aOR: 0.48 [95 %CI 0.16 – 1.44], p = 0.188) (Table 3). Maternal mortality was low in both the trained and not trained groups (0.4 % vs. 0.0 %, p=0.365) (Table 2). aHBB training was associated with a decreased odds of the composite outcome of neonatal subjective morbidity (neonatal ill health combined with mortality) (aOR: 0.31 [95 %CI 0.14 – 0.70] p=0.005) compared to the not trained group after adjusting for multiple gestations and birth attendant present at delivery. There was no difference in the composite outcome of maternal subjective morbidity. Assessing other measures of optimized care at delivery and health outcomes, there was no association between supplies ready at birth (p=0.900), birth attendant at delivery (p=0.575), and self-report of attending all aHBB trainings (p=0.168) and neonatal mortality (Table 4).

**Table 3:** aHBB Training and Health Outcomes (Mortality and Poor Health)

	Unadjusted		Model 1		
	**uOR**	**95 %CI**	**aOR**^**b**^	**95 %CI**	**p-value**
**Primary Outcome**					
Neonatal Mortality	0.55	0.25 – 1.21	0.48	0.16 – 1.44	0.19
**Secondary Outcome**					
Maternal Mortality	^a^		^a^		
Neonatal Ill Health/Mortality	0.49	0.28 – 0.87	0.31	0.14 – 0.70	0.005
Maternal Ill Health/Mortality	0.84	0.42 – 1.69	1.00	0.36 – 2.81	1.00

^a^Could not be estimated due to low number of cases and both cases being in the same exposure group (trained)

^b^Adjusted for multiple gestation (twins) and birth attendant present at delivery

**Table 4:** Optimized Delivery Care and Health Outcomes (Supplies at Birth, # of trainings, # births attended, and birth attendant at delivery)

	uOR	95 %CI	aOR^b^	95 %CI	p-value
*Supplies Ready at Birth*					
**Primary Outcome**					
Neonatal Mortality	0.42	0.16 – 1.08	0.82	0.04 – 17.10	0.90
**Secondary Outcome**					
Maternal Mortality	^a^		^a^		
Neonatal Ill Health/Mortality	0.79	0.34 – 1.87	1.13	0.11 – 12.09	0.92
Maternal Ill Health/Mortality	0.92	0.34 – 2.45	0.77	0.05 – 12.40	0.86
*Birth Attendant at Delivery*					
**Primary Outcome**					
Neonatal Mortality	0.48	0.20 – 1.17	0.50	0.04 – 5.66	0.58
**Secondary Outcome**					
Maternal Mortality	^a^		^a^		
Neonatal Ill Health/Mortality	0.82	0.38 – 1.77	3.57	0.42 – 30.32	0.24
Maternal Ill Health/Mortality	0.86	0.36 – 2.01	1.10	0.13 – 9.78	0.93
*All aHBB Trainings*					
**Primary Outcome**					
Neonatal Mortality	0.86	0.41 – 1.82	0.41	0.12 – 1.45	0.17
**Secondary Outcome**					
Maternal Mortality	^a^		^a^		
Neonatal Ill Health/Mortality	0.69	0.40 – 1.19	0.54	0.19 – 1.54	0.25
Maternal Ill Health/Mortality	0.76	0.42 – 1.39	0.29	0.09 – 0.96	0.04

^a^ Could not be estimated due to low number of cases and both cases being in the same exposure group (trained)

^b^ Adjusted for each of the factors listed above plus multiple gestation and HBB training

### Sensitivity analyses

When the definition of aHBB training was redefined to include (1) only those attending all three sessions or (2) any training (self-reported or attendance at one or more training sessions), there was no association between the redefined aHBB training status and neonatal mortality or other health outcomes (Table S2).

## Discussion

Neonatal mortality is an important health concern with a continued need to find innovative strategies to reduce global neonatal mortality. HBB has been shown in several studies to be associated with a reduction of neonatal mortality in clinical settings when delivered by trained birth attendants [4, 12]. Nevertheless, for a variety of systemic and cultural reasons many women still deliver at home, where neonatal resuscitation programs are not traditionally used. There is minimal guidance on how to improve neonatal health in populations that do not have access to a trained birth attendant. Focusing on improving care to mothers and neonates who do not have access to trained birth attendants may help to reduce the disparities found in neonatal mortality.

Our modified approach to the HBB was designed to assist women who are giving birth outside medical care facilities with no skilled birth attendants. Due to the fact that we were training laypeople to deliver care and could not provide the clinical training necessary to safely and appropriately use BVM, the aHBB program used in our community populations excluded BVM and advanced lifesaving techniques from the program [9, 13]. Based on new work by Kc et al., we might infer that 1.6 % (8.5 of 536) of the neonates in this population may have benefited from bag-valve-mask resuscitation [14]. However, improper bag-valve-mask resuscitation can cause pneumothoraces, poor venous return, and increases the risk of aspiration, and these risks increase without adequate training and practice [15]. In ICU settings, bag-valve-mask ventilations increase the odds of pneumothorax by 9-30 fold compared to controls [16, 17]. The risks and benefits of bag-valve-mask ventilation in the hands of relatively untrained providers deserves further discussion.

This study has several limitations. First, this study was not powered to find a difference in neonatal mortality. We observed a 4.5 % crude difference in neonatal mortality between the two groups and would not be able to conclude that this is truly no difference in mortality or if it is due to low statistical power from small sample size. Our data is similar to other larger studies done in low resource settings like Bangladesh [18] and Pakistan [19], but did not show as robust a difference as work done in Zambia [20]. Perhaps this is due to the size of these other studies, national differences in neonatal and maternal mortality rates at baseline, the exclusion of BVM, or the fact that this intervention focused on laypeople instead of health workers who have already had some training [21]. This is difficult to determine because of the range of training and experience that village health workers in different settings and different studies possess [22]. As differences in the composite outcome of neonatal subjective morbidity were observed indicating a benefit of aHBB training, an important future study would be to identify if there is a difference in neonatal mortality with a larger study population in the Haitian context.

Second, there was an overall low number of neonatal and maternal deaths. This could indicate that the group of women accessing the pop-up clinics where the study sample was recruited have better baseline health status than the average Haitian women. If this is true, the effect of aHBB training could be understated in this study.

Third, there was missing data in this study cohort, including exposure and outcome. We did compare the participant characteristics of the complete case analysis study cohort with those that were missing data and did not find any significant differences. However, there could still be differences in unmeasured characteristics that could bias the study results.

Another contribution to the modesty of these results may be due to the variable period of time between birth and follow up by CHWs. There are some studies to suggest that implementation of HBB decreases early neonatal mortality, but that the impact on mortality is gone within 6 days [23]. Wrammert et al. postulate that this is because the neonates succumbed to infection, hypothermia, and other conditions when HBB was taught without attention to other aspects of neonates care. The fact that there was a variable interval between birth and follow up may have obscured some of the benefit from the program.

Finally, this study included a few important biases. The fact that the CHWs were not blinded to exposure to aHBB training may have resulted in observer bias and affected the results. Additionally, since the CHWs are the ones enrolling people in the study these results are vulnerable to selection bias, as the CHWs are more likely to enroll people who they know and think will be cooperative. Participants who elected to be a part of the study may be more engaged than those who did not elect to participate.

Reduction in neonatal subjective morbidity, as described by the mothers, was statistically significant. This measure was a composite that included neonates described as ill and dead. Several studies in high income countries have shown that parental concern for ill health is a significant predictor of serious illness [24]. In the aHBB group, mothers were more likely to have live babies that they felt were healthy and were also more likely to have had a birth attendant present with supplies ready at time of birth. This may have played a role in reduction of neonatal subjective morbidity, but not mortality alone. The ability to reduce morbidity and mortality in neonates supports global efforts to improve health in children under the age of five. More research is needed to see if healthier neonates could influence under five childhood mortality, which remains high in Haiti at 71.7 per 1000 live births in 2019 [25].

This study shows that it is feasible to train laypeople with variable amounts of background education using a modified HBB curriculum. Our work shows that those lay people can use the training to improve the chance that a trained person with proper supplies is present at birth, even in an isolated population who cannot easily access traditional healthcare. Furthermore, with this appears to have a positive impact on the composite outcome of neonatal morbidity and mortality.

Future studies may build upon this work by scaling it to a larger population to adequately assess for a significant difference in neonatal mortality. Additionally, separating the jobs of performing the training and collecting the data into different groups would mitigate some of the bias in this study. We feel that this work provides enough evidence to show that an aHBB program can be established when there are significant barriers to traditional health care and access to trained birth attendants is limited.

## Conclusions

Our study results are encouraging that a reduction in neonatal subjective morbidity can be achieved with a simplified version of HBB that is taught to laypersons. To new mothers, having a child who they feel is not sick is an important patient-centered outcome. As our global community strives to reduce neonatal mortality and tackle disparities in neonatal mortality, an aHBB program is one tool that can be used in low-resource settings where skilled birth attendants are limited. Moreover, the aHBB program may be used during home births where mothers face challenges in obtaining obstetric care in a healthcare setting.

## Supplementary Information

**Additional file 1.**

## Data Availability

The datasets used and/or analyzed during the current study are available from the corresponding author on reasonable request.

## Abbreviations

HBB: Helping Babies Breathe
aHBB: adapted HBB
SDG: Sustainability Development Goals
WHO: World Health Organization
AAP: American Academy of Pediatrics
USAID: United States Agency for International Development
NICHD: the National Institute of Child Health and Human Development
BVM: bag-mask-ventilation
CHWs: Community Health Workers
