# Supplementary material for: Adapted Helping Babies Breathe approach to neonatal resuscitation in Haiti: a retrospective cohort study
Source: BMC Pediatr. 2022 Jan 3;22:7. doi: 10.1186/s12887-021-02987-4 (PMC8722303; doi:10.1186/s12887-021-02987-4)
Supplement: Supplementary file 1 — Additional file 1. [file 12887_2021_2987_MOESM1_ESM.docx]

**Supplemental Tables**

Table S1. Demographics of Respondents in Analysis Population compared to those with missing training status or lost-to-follow-up.

|  | **Study Cohort** |  | **Missing Data** |  |  |
| --- | --- | --- | --- | --- | --- |
|  | **N= 536** |  | **N= 244** |  |  |
| Variable | n | % | N | % | p-value* |
| **Singleton vs Multiple Gestation Birth** |  |  |  |  | 0.09 |
| 1 | 496 | 92.5 | 105 | 43.0 |  |
| 2 | 12 | 2.2 | 3 | 1.2 |  |
| Missing | 28 | 5.2 | 136 | 55.7 |  |
| **Births Attended**, median (IQR) | 0 | (0-2) | 0 | (0-2) | 0.28 |
| **Birth Attendant at Delivery** |  |  |  |  | 0.59 |
| Yes | 450 | 84.0 | 14 | 5.7 |  |
| No | 68 | 12.7 | 3 | 1.2 |  |
| Missing | 18 | 3.4 | 227 | 93.0 |  |
| **aHBB Supplies ready on time** |  |  |  |  | 0.48 |
| Yes | 450 | 84.0 | 6 | 2.5 |  |
| No | 43 | 8.0 | 1 | 0.4 |  |
| Missing | 43 | 8.0 | 237 | 97.1 |  |
| **Neonatal Health** |  |  |  |  | 0.68 |
| Good Health | 456 | 85.1 | 101 | 41.4 |  |
| Ill Health | 40 | 7.5 | 6 | 2.5 |  |
| Dead | 37 | 6.9 | 9 | 3.7 |  |
| Unknown | 3 | 0.6 | 128 | 52.5 |  |
| **Maternal Health** |  |  |  |  | 0.64 |
| Good Health | 469 | 87.5 | 104 | 42.6 |  |
| Ill Health | 56 | 10.5 | 11 | 4.5 |  |
| Dead | 2 | 0.4 | 1 | 0.4 |  |
| Unknown | 9 | 1.7 | 128 | 52.5 |  |

*Does not include missing/unknown categories

Table S2. Sensitivity Analysis Results

|  | **Sensitivity Analysis 1*** | | |
| --- | --- | --- | --- |
|  | **aOR**** | **95%CI** | **p-value** |
| **Primary Outcome** |  |  |  |
| Neonatal Mortality | 0.96 | 0.47-1.96 | 0.91 |
| **Secondary Outcome** |  |  |  |
| Maternal Mortality | 0.68 | 0.04-11.00 | 0.79 |
| Neonatal Ill Health/Mortality | 0.64 | 0.38-1.07 | 0.09 |
| Maternal Ill Health/Mortality | 0.61 | 0.34-1.08 | 0.09 |
|  | **Sensitivity Analysis 2**** | | |
|  | **aOR**** | **95%CI** | **p-value** |
| **Primary Outcome** |  |  |  |
| Neonatal Mortality | 3.33 | 0.42-26.43 | 0.26 |
| **Secondary Outcome** |  |  |  |
| Maternal Mortality | * |  |  |
| Neonatal Ill Health/Mortality | 0.77 | 0.29-2.02 | 0.59 |
| Maternal Ill Health/Mortality | 0.84 | 0.27-2.62 | 0.77 |

*Sensitivity analysis 1 redefined aHBB training as recorded attendance at all three aHBB training sessions (regardless of self-report status).

**Sensitivity analysis 2 defined aHBB training as not trained meaning no training and self-report status of no training. All other subjects were considered trained.
